# Structure of Turnip mosaic virus and its viral-like particles

**DOI:** 10.1038/s41598-019-51823-4

**Published:** 2019-10-28

**Authors:** Rebeca Cuesta, Carmen Yuste-Calvo, David Gil-Cartón, Flora Sánchez, Fernando Ponz, Mikel Valle

**Affiliations:** 1 Molecular Recognition and Host–pathogen Interactions Programme, CIC bioGUNE, Bizkaia Technology Park, 48160 Derio, Spain; 2 Centro de Biotecnología y Genómica de Plantas, Universidad Politécnica de Madrid - Instituto Nacional de Investigación y Tecnología Agraria y Alimentaria (CBGP, UPM-INIA), Campus Montegancedo, 28223 Madrid, Spain

**Keywords:** Virus structures, Cryoelectron microscopy

## Abstract

*Turnip mosaic virus* (TuMV), a potyvirus, is a flexible filamentous plant virus that displays a helical arrangement of coat protein copies (CPs) bound to the ssRNA genome. TuMV is a bona fide representative of the Potyvirus genus, one of most abundant groups of plant viruses, which displays a very wide host range. We have studied by cryoEM the structure of TuMV virions and its viral-like particles (VLPs) to explore the role of the interactions between proteins and RNA in the assembly of the virions. The results show that the CP-RNA interaction is needed for the correct orientation of the CP N-terminal arm, a region that plays as a molecular staple between CP subunits in the fully assembled virion.

## Introduction

Flexible filamentous viruses are plant pathogens that cause important reduction in crop yields and comprise about four hundred different species distributed in four families: *Alphaflexiviridae*, *Betaflexiviridae*, *Closteroviridae*, and *Potyviridae* (https://viralzone.expasy.org/751). Their virions are long (hundreds of nm), thin (about 13–15 nm in diameter) and flexible, and contain a monopartite (+)ssRNA genome covered by hundreds of subunits of the CP organized in helical fashion^1^. Recent cryoEM studies have solved the structure of several representatives of these helical viruses. The structures for *Bamboo mosaic virus* (BaMV)^2^ and *Pepino mosaic virus* (PepMV)^3^, two potexviruses, together with the structure of potyviruses *Watermelon mosaic virus* (WMV)^4^ and *Potato virus Y* (PVY)^5^, have shown that these elongated virions display the identical left-handed helical arrangement, and that their CPs share the same fold^4,6^ despite the lack of sequence homology between CPs of viruses from different families.

There is growing interest in the use of plant viruses for nanobiotechnological purposes^7^, specially in biomedical applications where the low potential risk of plant viruses for mammals is a clear advantage^8^. Elongated flexible viruses with helical symmetry can be modified at the level of their CP by genetic engineering or chemical conjugation^9^, and can be used for delivery, imaging, and theranostics purposes. Virions with introduced peptides in their CPs present the antigen in a repetitive and symmetrical way, and it has been shown that they serve as efficient vaccine platforms^10,11^. Virus like particles (VLPs) devoid of the viral genome are also good nanobiotechnological tools. VLPs of flexible filamentous plant viruses have been produced by the heterologous expression of CPs in bacteria, yeast, insect cells and plants^12^. The biotechnological use of viral nanoparticles (VNPs), which include viruses and VLPs, relies on the successful design of genetic or chemical modifications^13^. Structural information about VLPs from flexible filamentous plant viruses has been scarce^14^, but recently a high resolution study for VLPs from PVY^5^ has shown that the filaments are assembled from octameric rings of the CP, i. e., a nonhelical organization. In this work we explore the structure of TuMV virions and VLPs to unveil the differences in their architecture and understand the contribution of protein-RNA interactions in the assembly of the virions. We observe that TuMV VLPs produced in plants conserve the helical architecture of the virion and that the absence of the ssRNA precludes the interaction between CP subunits mediated by the N-terminal arm.

## Results and Discussion

**Figure 1:**
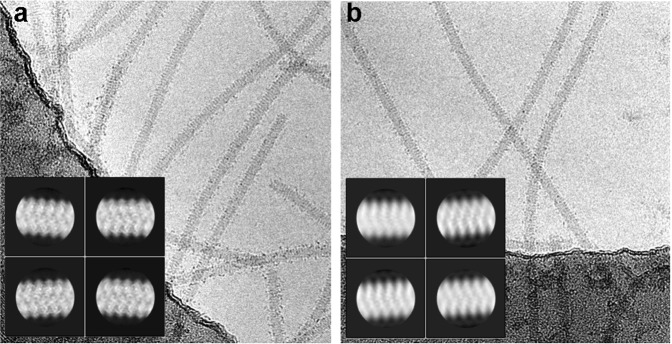
CryoEM imaging of TuMV virions and TuMV VLPs. Panels show cryoEM images for TuMV virions (**a**) and TuMV VLPs (**b**). The insets display representative 2D averages for both samples after reference-free classification.

Using cryoEM and following single particle-based helical image processing, we have explored the structure of the potyvirus TuMV and its VLPs. TuMV virions were isolated from infected plants of Indian mustard, and VLPs of TuMV CP were produced by its transient expression in *Nicotiana benthamiana* plants^15^. Filaments of virions (Fig. 1a) and VLPs (Fig. 1b) look very similar in cryoEM images, although the VLPs are more variable in length^13^. Extracted segments of the filaments were aligned and classified, and the 2D averages for TuMV virions and TuMV VLPs are significantly different (insets in Fig. 1). The aligned viral segments display averages with high resolution information with local details attributable to the projection of secondary structural elements of the CPs. The averages from TuMV VLPs, however, are blurred and suggest the presence of structural heterogeneity. These images do not display any pattern of parallel densities, thus, do not suggest TuMV VLPs constructed by stacked rings.

**Figure 2:**
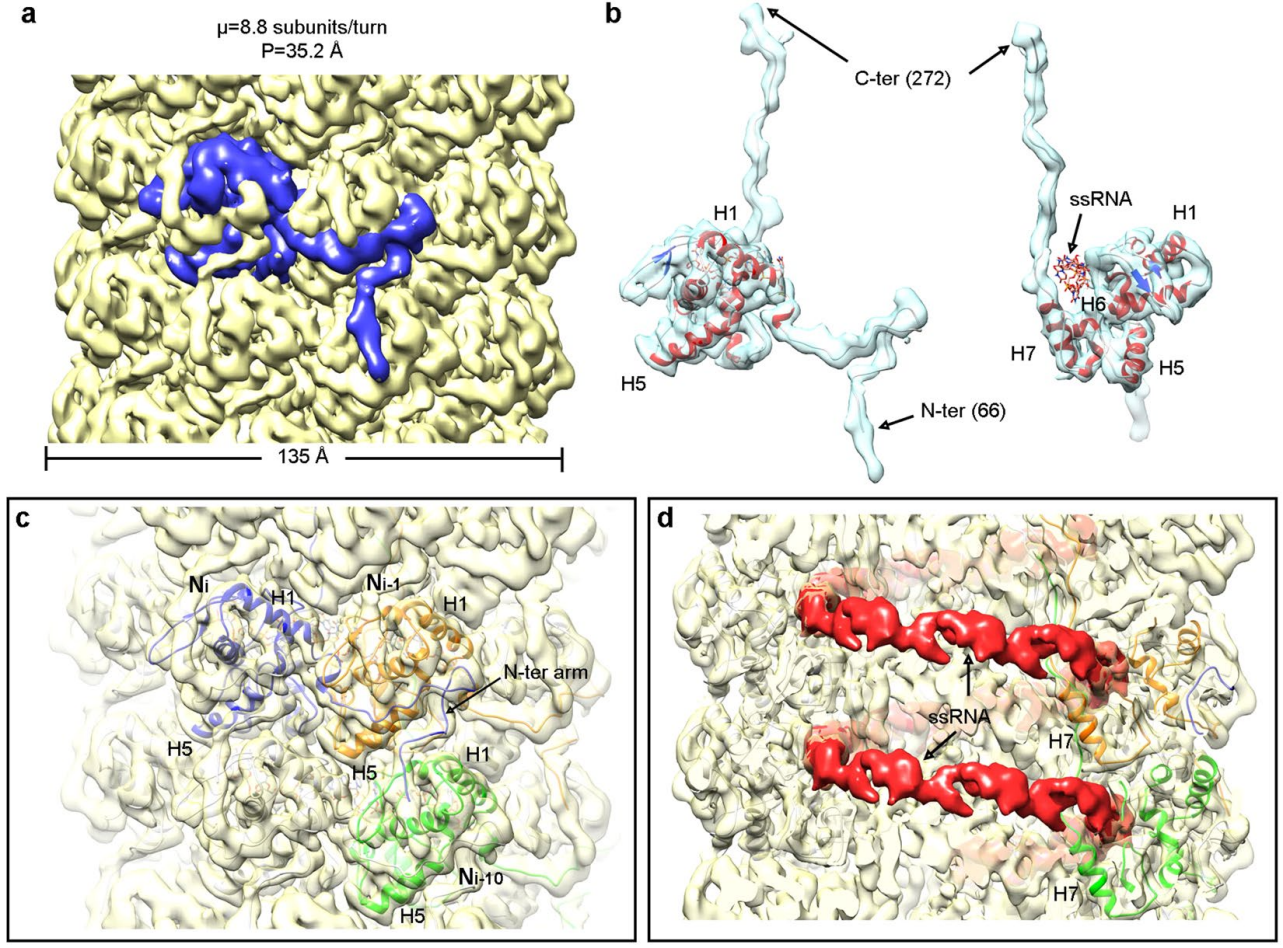
CryoEM 3D structure of TuMV. (**a**) Rendering of the 3D map calculated for TuMV virions (yellow). The density for one of the CP subunits is depicted blue. Helical symmetry parameters are indicated: µ stands for the number of subunits per turn of the helix; and P for the helical pitch. (**b**) Semitransparent representation of the density attributed to a single TuMV CP, together with the modeled atomic coordinates and a polyU that represent the ssRNA. Two different orientations are shown. (**c**) The cryoEM map for TuMV is seen semitransparent together with the fitted structures for several CP subunits displayed in different colors. (**d**) Cut-away view of TuMV cryoEM map with ribbons from the fitted coordinates for some CP subunits. The isolated density for the ssRNA is seen in solid mode and red colored. Along the panels some α-helices of the atomic structure for TuMV CP are labeled (H1, H5, H6, and H7).

The cryoEM 3D map for TuMV virions (Fig. 2a) shows a left-handed helical arrangement identical to that of earlier characterized flexible filamentous plant viruses^2–5^. Unsupervised 3D classification of the total data set for TuMV virions reveals that regions of the filaments stretch and shrink with an amplitude of around 2 Å per turn (Supplementary Fig. 1a–c and Movie M1). This flexibility of the virions might have limited the resolution which is estimated at approximately 5 Å for the three classes. We have used the 3D map for the most populated group (Supplementary Fig. 1b) for the calculation of the atomic model for TuMV CP. As mentioned earlier, the 3D fold of the CPs from flexible filamentous viruses of different families is highly conserved^2–4,6^ despite the absence of sequence homology between them. Within potyviruses the known CP structures for WMV^4^ and PVY^5^ are almost identical, with rmsd value between Cα atoms around 2 Å. The CP from TuMV shows high sequence conservation with these both CPs. Thus, we expect the structure of TuMV to be alike to the structures for the two other potyviruses, WMV and PVY. Actually, the 3D cryoEM maps for TuMV, WMV, and PVY superimpose in almost full agreement (a comparison with WMV is shown in Supplementary Fig. 1e,f). Even though our cryoEM map for TuMV is limited to 5 Å resolution, the high sequence homology and structural conservation allow us to build an accurate atomic model for TuMV CP (Supplementary Fig. 2) based on the structure for WMV CP (pdb code 5ODV)^4^. The sequence homology between the two nucleoproteins is of 63% identities and 80% positives in the modeled region. The atomic coordinates for TuMV CP show a central alpha-helical core and two long arms (Fig. 2b). The cryoEM map does not show density (we could not model them) for the first 65 amino acids at the N-terminal end, a flexible region exposed to the solvent. In this regard, cryoEM images for both, virions and VLPs, show small electron-dense bodies around the filaments (Fig. 1) suggesting the presence of partially folded and globular domains in this flexible N-terminus of TuMV CP. The last 16 residues at the C-terminus cannot be traced. As shown before^2–4^, the participation of flexible N- and C-terminal arms in the interaction between CP subunits is the structural basis for the flexible nature of the virions. The N-terminal arm of each TuMV CP interacts with other two subunits (Fig. 2a,c). There is a side-by-side interaction between the N-terminal arm and a groove in the adjacent subunit mediated by hydrophobic interactions (Fig. 2c and Supplementary Fig. 2b). After a 90° turn, the N-terminal arm reaches another subunit in the next turn of the helix where the interaction is favored by complementary electrostatic potentials (Fig. 2c and Supplementary Fig. 2b). The dual role for the N-terminal arm that supports side and axial polymerization and the nature of the local interactions (hydrophobic and electrostatic) were also observed for WMV^4^ and PVY^5^ and seem to describe a signature for potyviruses. The density for the ssRNA is clear (red density in Fig. 2d) and each TuMV CP subunit spans five nucleotides of the viral genome. The ssRNA stands in a groove at the folded central domain, just next to the last helix H7 (Fig. 2b), and the RNA binding site of TuMV CP includes the universally conserved pocket in flexible filamentous plant viruses formed by amino acids Ser, Arg, and Asp (Supplementary Fig. 2c)^4,6^.

For TuMV VLPs initial cryoEM results imposing helical symmetry did not converge in reproducible 3D maps (data not shown), thus, a 3D classification of the filament segments was performed without any imposed symmetry. The results (Supplementary Fig. 3) revealed that only about 60% of the particles display clear helical arrangement with well defined CP subunits (classes 1 and 3 in Supplementary Fig. 3a,c), while the rest of the groups show 3D maps with poor structural features and no indication of well ordered helical arrangement (Supplementary Fig. 3b,d–f). Thus, the absence of ssRNA in the VLPs produces labile multimers with distorted local regions along the filaments. This classification did not detect any population of VLPs constructed by stacked rings.

**Figure 3:**
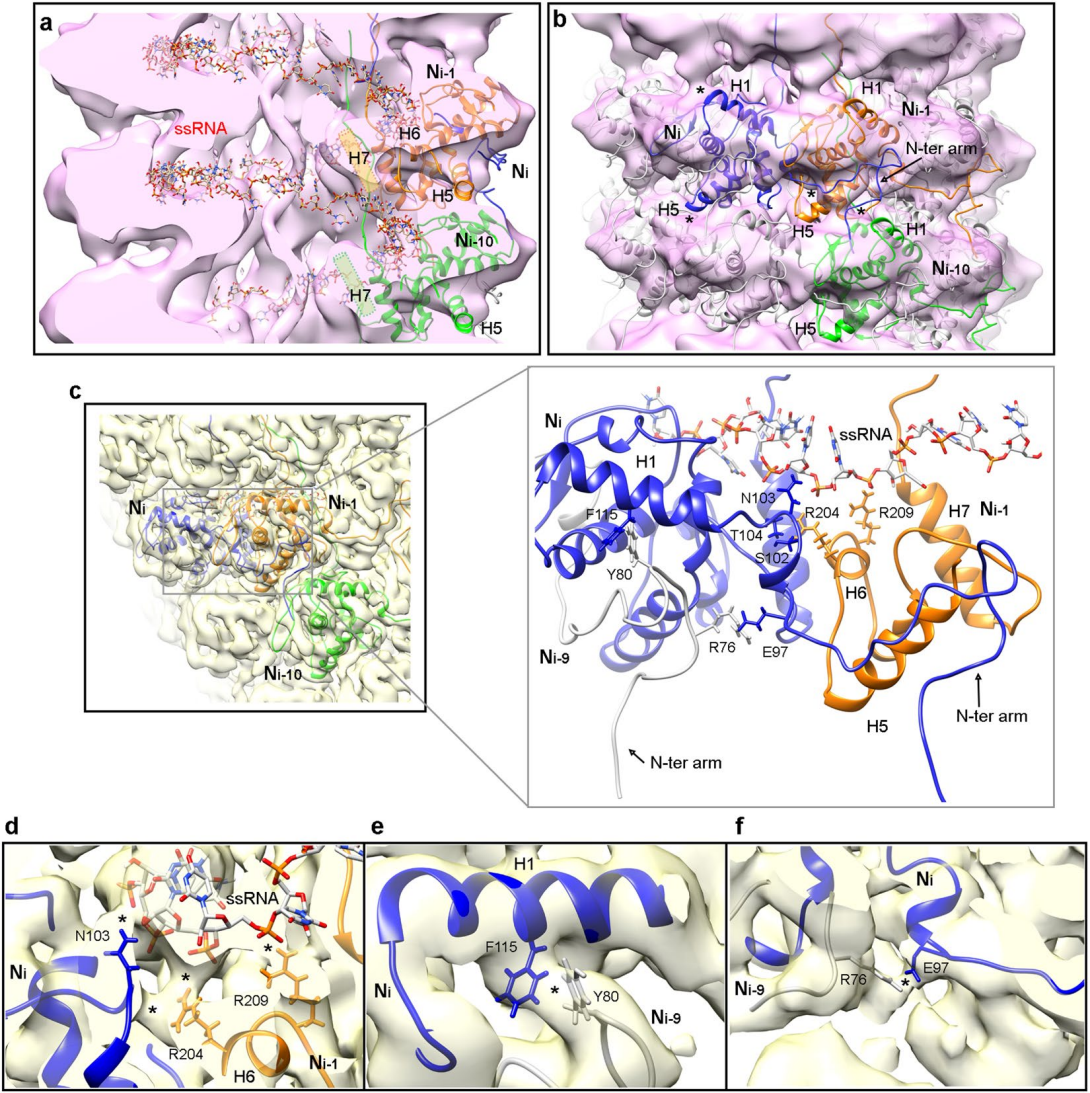
Structure of VLPs and the role of CP-RNA interactions. (**a**) Cut-away rendering of the cryoEM map for class 1 of TuMV VLP. Atomic models for several TuMV CPs and the ssRNA are also included. After rigid body fitting of the coordinates derived from TuMV virion, the ssRNA runs in an empty channel. The position of helix H7 seem to have moved in the VLP towards the inner side of the filament, and the new putative location is indicated by cylinders. (**b**) The fitted coordinates for the multimer of TuMV CPs are seen inside the semitransparent map for class 1 of TuMV VLP. Regions of the atomic models that lie outside the density are labeled with asterisks in subunit N_i_. (**c**) Protein-RNA and protein-protein interactions at the interface between CP subunits in TuMV virions. Three CP subunits are depicted, together with the ssRNA. Residues that participate in protein-RNA and/or protein-protein interactions are indicated. Some regions of CP subunit N_i-1_ are not displayed for clarity. The thumbnail at the left shows the orientation. (**d**–**f**) Close-up views of the cryoEM map and atomic coordinates for TuMV virions focused on the regions of protein-protein and protein-RNA interactions. The contacts between residues (labeled with asterisks) are visible in the 3D density map at 2σ (panels d,e) or 1σ density thresholds. In the panels some α-helices of the atomic structure for TuMV CP are labeled.

The two groups of VLP segments with good helical features (classes 1 and 3) were further refined to 3D maps with final resolutions about 8 Å (Supplementary Fig. 4). This poor definition compared with the results for TuMV virions, suggests that VLPs are less stable, structurally more heterogeneous, and hence their 3D averages are limited in structural details. At this level of resolution it is not possible to build accurate atomic models. Both groups, however, exhibit helical symmetry parameters (Supplementary Fig. 4) identical to that of the TuMV virions (Fig. 2), thus, we assume that the overall organization of the virions is kept in the VLPs despite the lack of nucleic acid. For the interpretation of the structures for VLPs, we fit the atomic coordinates modeled for TuMV virions (a polymer of 20 CP subunits) as a rigid body. In the cryoEM maps for both groups of VLP segments, the helical path for the ssRNA derived from TuMV virions (the atoms for the nucleic acid were not included in the rigid body fitting) resides in an empty passage (Fig. 3a and Supplementary Fig. 5a). This confirms the absence of the ssRNA in the VLPs and that the fitting of the CP multimer is on the correct register with respect to the 3D maps. In class 1, helix H7, that delimits the ssRNA binding groove in the virions (Fig. 2d), seems to move towards the inner side of the filament (Fig. 3a). The fitting of the coordinates for the oligomer of CPs lefts the N-terminal arm outside the density: fully outside in class 1 (Fig. 3b); or only in the last region that participates in axial interactions in class 3 (Supplementary Fig. 5b). Also, the densities for helices H1 and H5 are incomplete, and both secondary structure elements stick out at certain degree from the cryoEM maps (Fig. 3b and Supplementary Fig. 5b). Thus, the role of the N-terminal arm in polymerization and the position of helices H1 and H7 are perturbed in the absence of the ssRNA. To gain some insights into the influence of the ssRNA over these structural elements we revisit the atomic model for TuMV virions (Fig. 3c). In the boundary between CP subunits there is a network of protein-RNA and protein-protein interactions that supports the proper orientation of the flexible N-terminal arm. Residue N103 from one CP subunit (N_i_), and the pair R204 and R209 from the adjacent CP (N_i-1_) interact with the phosphate backbone of the ssRNA (Fig. 3d). At the same time, these two regions are connected between them, in such a way that R204 interacts with the beginning of the N-terminal arm that contains the aforementioned N103, and S102 and T104 at the neighboring subunit (Fig. 3c). These local interactions with the ssRNA and between CPs serve to anchor helix H1 and the N-terminal arm of one CP subunit (N_i_) and helices H5 and H6 of the neighbor (N_i-1_). Since helix H5 builds part of the groove for the interaction with the N-terminal arm, the contacts with the ssRNA modulate both the donor and the acceptor in the interaction via the N-terminal arm. The three residues that make direct contact with the ssRNA in this region are highly conserved in potyviruses (N103 90%, R204 80%, and R204 83%) and are also seen involved in the same interactions with the nucleic acid in WMV^4^ and PVY^5^. In this same local region, helix H1 and the N-terminal arm (subunit N_i_) interact with the N-terminal arm of other subunit from the next helical turn (N_i-9_ in Fig. 3c). Here, the hydrophobic interaction F115-Y80 (Fig. 3e) and the salt bridge E97-R76 (Fig. 3f) are key to set the 90° turn of the N-terminal arm towards the next turn of the helix. The F115-Y80 connection between TuMV CPs has equivalent pairs in WMV and PVY, where the hydrophobic pair is established between Tyr and Val residues. However, the E97-R76 salt bridge has no counterparts in the other two potyviruses, probably due the high diversity of sequences at the N-terminal arm.

As opposed to icosahedral viruses, in helical viruses the genetic material is bound to copies of the viral nucleoprotein or CP along the entire genomic length, and each nucleoprotein subunit interacts with the genome. Thus, the absence of the nucleic acid in VLPs is expected to modify the entire structure. Interestingly, the VLPs in the current work keep the helical symmetry of the virions, while PVY VLPs derived from overpexpressed CP subunits in *E*.*coli* arrange in the form of stacked rings of 8 subunits. Although at lower resolution, VLPs from *Alternanthera mosaic virus* (AltMV, a potexvirus) produced *in vitro* were seen in helical arrangement^14^. These differences in the architecture of VLP assemblies need to be further explored for the design of nanoparticles based on CPs from flexible filamentous plant viruses. The helical arrangement of TuMV VLPs allows the comparison of their structure with TuMV virions, and shows that the interaction with the ssRNA in between subunits govern the network of contacts between CPs mediated by N-terminal arms that play as molecular staples, and that these interactions are lost in the absence of the nucleic acid.

## Materials and Methods

### Purification of TuMV virions and VLPs

TuMV (isolate UK 1)^16^ was propagated in plants of Indian mustard (*Brassica juncea*), which were harvested 30 days post-inoculation. For VLP production, five-week old *Nicotiana benthamiana* plants were agroinfiltrated for CP transient expression. *Agrobacterium tumefaciens* (LBA4404 strain) transformed with the CP construction was subcultured and grown overnight, pelleted at 2000 × *g*, resuspended to OD_600_ = 1.2 in MMA buffer (10 mM MES, pH 5.6; 10 mM magnesium chloride; 450 µM acetosyringone), and then infiltrated into the leaves using a blunt-ended 2 mL syringe. Tissue was harvested 10–12 days post-agroinfiltration. VNPs were purified either from Indian mustard (150 g) or *N*. *benthamiana* (100 g) plant material as described^10^. Briefly, plant tissue was finely ground in 0.5 M potassium phosphate pH 7.5, 1:2 (w/v) in an electrical tissue grinder, at 4 °C. The resulting suspension was extracted with one volume of chloroform at 4 °C. Phases were separated by centrifugation; aqueous phase was filtered through Miracloth. After that, VNPs were precipitated with 6% PEG 6000 (w/v), 4% NaCl (w/v). They were allowed to precipitate for 90 min at 4 °C. The particles were recovered by centrifugation for 10 min at 12,000 × g. The pellet was resuspended overnight in 0.5 M potassium phosphate pH 7.5, 10 mM EDTA. The solution was clarified by centrifugation (10 min at 9 000 × g) and the VNPs pelleted (2 h at 80 000 × g). The pellet was resuspended in 0.25 M potassium phosphate pH 7.5, 10 mM EDTA, and CsCl was added to a final density of 1.27 g/cm^3^. The resulting solution was subjected to centrifugation at 150 000 × g for 18 h at 4 °C. A visible band in the gradient containing the particles was recovered by punching the tube with syringe and needle. It was diluted in 0.25 M potassium phosphate pH 7.5, 10 mM EDTA and pelleted by centrifugation (2 h at 80 000 × g). Finally, the pellet was resuspended in 50% glycerol (v/v), 5 mM Tris pH 7.5, 5 mM EDTA at a final concentration of 1 mg/ml, and stored at −20 °C until further use. VNP concentration was determined spectrophotometrically considering an absorption coefficient (A_0.1%, 1 cm_ at 260 nm) of 2.65.

### CryoEM and image processing

TuMV VNPs solutions were applied to Quantifoil R2/2 holey carbon grids covered with a thin carbon layer, and the cryoEM grids were prepared using a Vitrobot (FEI). Movie frames images were collected in a Titan Krios FEI electron microscope operated at 300 kV using a K2 direct detector (GATAN). Nominal magnification was of x130.000 for both TuMV virions and VLPs, resulting in a sampling of 1.1 Å/pixel. The micrographs were taken during 9 s exposures in electron counting mode producing movies with 40 frames and a total dose of 40 e^−^/Å^2^. Motion between frames was corrected using frames 3 to 31, resulting in accumulative dose of 31 e^−^/Å^2^. The contrast transfer function of the micrographs for both samples was estimated using CTFFIND3^17^. In the corrected micrographs filaments were manually selected in Relion2^18^, resulting in 444,678 overlapping segments (boxes of 250 pixels × 250 pixels, with a step of 8 pixels between segments) for TuMV virions and in 307,333 segments (boxes of 200 pixels × 200 pixels, with a step of 8 pixels between segments) for TuMV VLPs. CryoEM density maps were calculated in Relion2. First, 2D classification and particle sorting allowed us to isolate sets of good-quality filament segments (335,228 segments for TuMV virions, and 118,597 for VLPs). For 3D refinement of TuMV virions, the initial reference was a cylinder with the diameter of the filaments. Starting with such featureless structure required to re-run the 3D refinement using the output from the first refinement as the new reference for a second round. Local optimization of twist and rise was carried out during 3D refinements. 3D classification with local searches of symmetry resulted in three classes with different, although very similar, symmetry values. Helical symmetry in real space was imposed to the final map to obtain homogeneity among the asymmetric units for molecular modeling. In the other hand, for TuMV VLPs the density map for TuMV virions low-pass filtered at 40 Å was used as initial reference map. Several 3D classifications were carried out, but only the one performed without imposing symmetry segregated classes with clear helical symmetry from distorted segments. Helical symmetry was imposed during the 3D refinement of selected classes and to their final 3D cryoEM maps.

### Atomic model building for TuMV CP

The atomic structure for WMV CP (pdb code 5ODV)^4^ was used as a template for the atomic model building of TuMV CP. Density for a TuMV CP subunit was isolated from the 3D cryoEM map by segmentation using the Segger^19^ method in Chimera^20^, which was also used to produce figures and movies. The amino acid sequence of WMW CP was mutated manually using Coot^21^ to match the sequence for TuMV CP. Further modeling of the TuMV CP atomic structure was carried out manually using Coot^21^ and the stereochemistry of the model was improved by real-space refinement in Phenix^22^. When the final atomic coordinates for TuMV CP are compared with the structure of WMV CP, the rmsd between their C-alpha backbone is just 1.2 Å in the modeled region. For the ssRNA, a modeled polyU was included in the refinement. To build up a multimer of CPs and the ssRNA a final refinement in Phenix was performed using noncrystallographic symmetry. Evaluation of the modeled atomic structure for TuMV CP was carried out in MolProbity^23^. To calculate the surface electrostatic potential for the atomic structure of TuMV CP, the atomic coordinates were loaded in Bluues server (http://protein.bio.unipd.it/bluues)^24^ and the potential was determined based on generalized Born radii^25^.

## Supplementary information

Supplementary Information

Supplementary Video M1
