# Supplementary material for: Structure of Turnip mosaic virus and its viral-like particles
Source: Sci Rep. 2019 Oct 28;9:15396. doi: 10.1038/s41598-019-51823-4 (PMC6817885; doi:10.1038/s41598-019-51823-4)
Supplement: Supplementary file 1 — Supplementary Information [file 41598_2019_51823_MOESM1_ESM.doc]

**Structure of Turnip mosaic virus and its viral-like particles**

Rebeca Cuesta, Carmen Yuste-Calvo, David Gil-Cartón, Flora Sánchez, Fernando Ponz, Mikel Valle

**Supplementary information**

**Supplementary Figures and Movie**


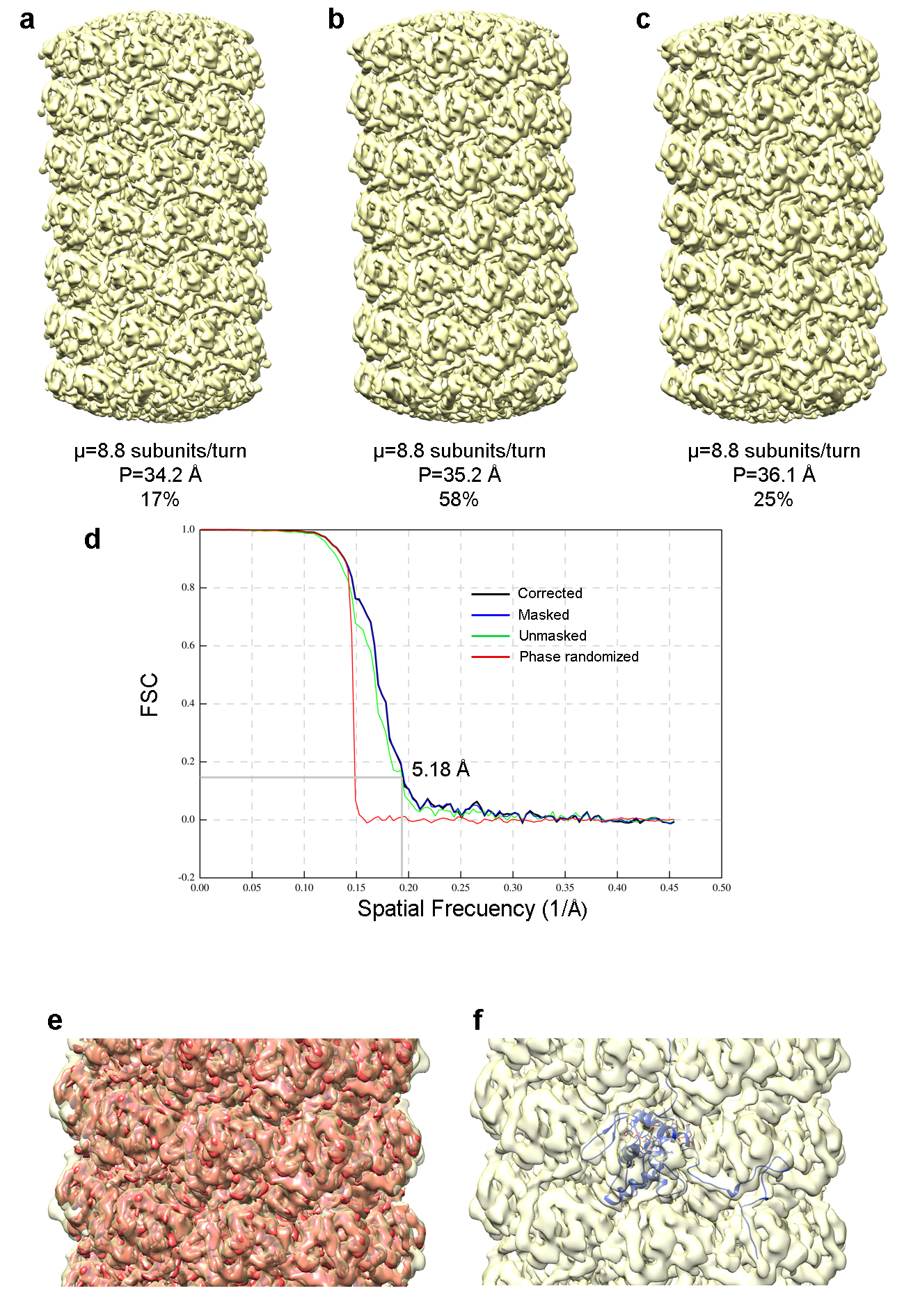


**Supplementary Fig.1 CryoEM maps for TuMV virions. a-c**, Rendering of the cryoEM maps for TuMV virions after classification of the total data set into three groups. Helical symmetry parameters and the fraction of the segments attributed to each group are indicated. **d**, Fourier Shell Correlation (FSC) after refinement and postprocessing of the cryoEM map for the most populated group of TuMV (class 2 shown in panel b). The FSC threshold at 0.14 estimates the resolution around 5 Å. **e** and **f**, overlay of the cryoEM map for TuMV virions (semi-transparent yellow) with the cryoEM map for WMW (e) or the fitted atomic coordinates for WMV CP (f).


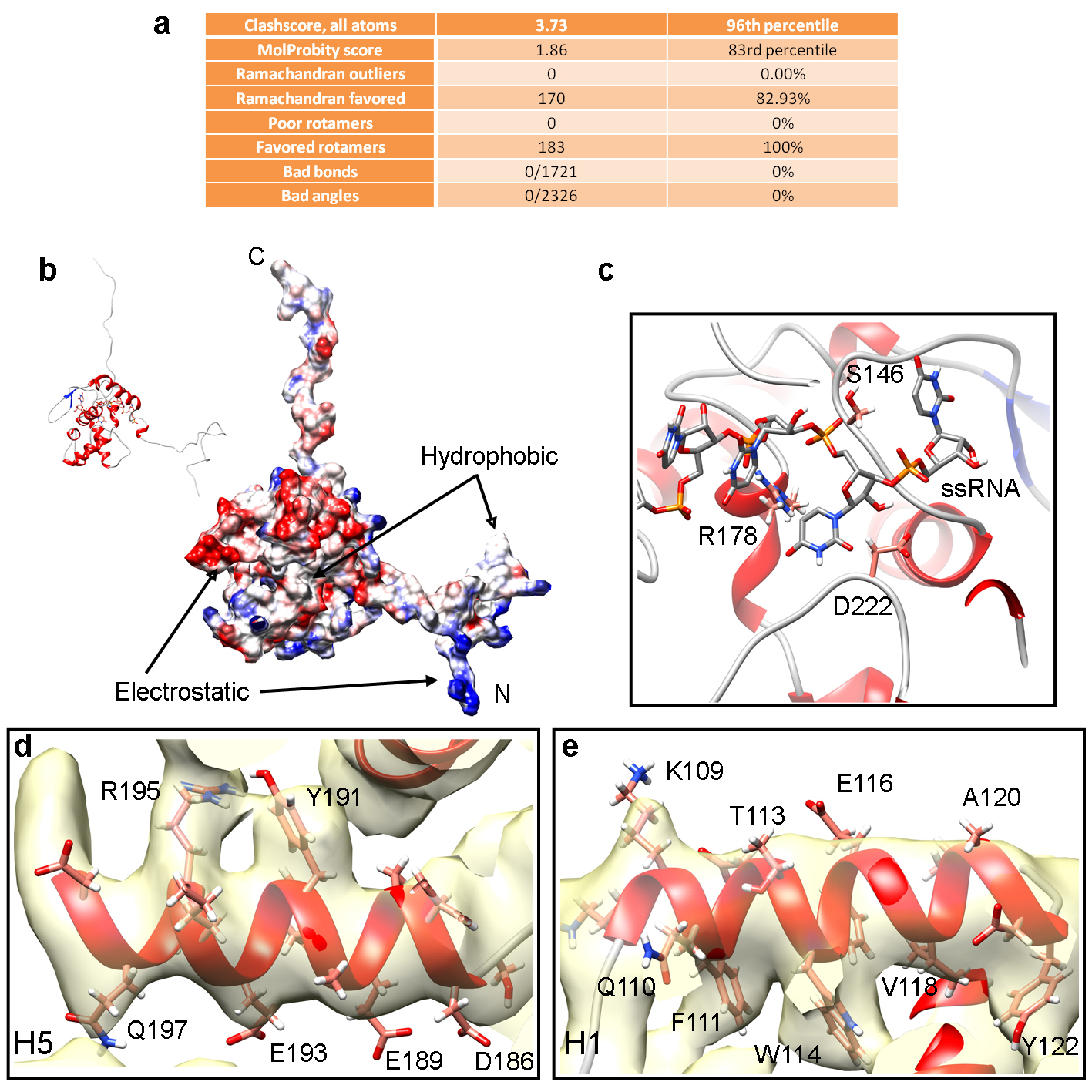


**Supplementary Fig.2 Atomic model for TuMV CP. a**, Parameters obtained in Molprobity that evaluate the stereochemistry of the atomic model for TuMV CP. **b**, Rendering of the electrostatic surface potential calculated for TuMV CP. Regions that participate in hydrophobic or electrostatic interactions mediated by the N-terminal arm are indicated. **c**, Region of the ssRNA binding site in TuMV CP that contains the universally conserved RNA binding pocket shown in all flexible filamentous plant viruses. Conserved amino acids Ser (S), Arg (R) and Asp (D) are displayed. **d** and **e**, Close-up views of the cryoEM map for TuMV virion together with the fitted atomic model for TuMV CP. The panels show σ-helices H5 (d) and H1 (e) where the position of side chains of amino acids are well seen in the 3D density map.


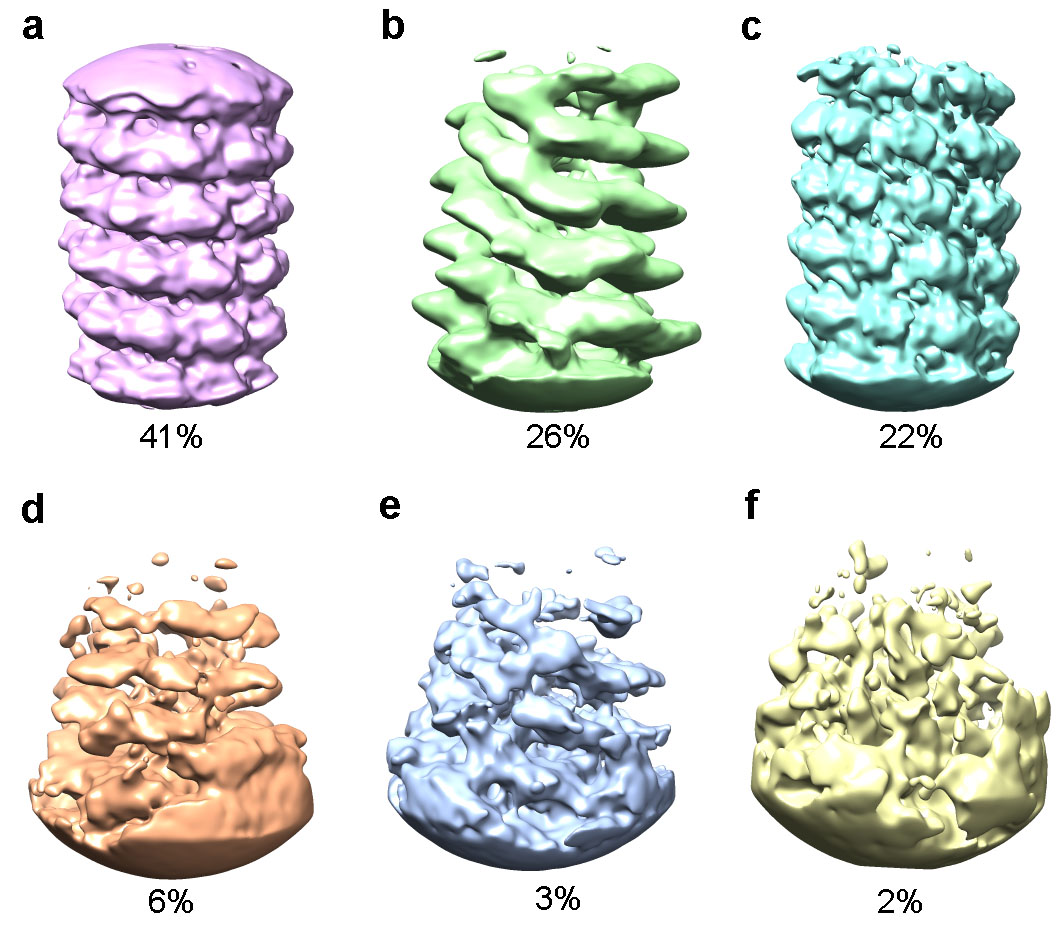


**Supplementary Fig.3 Classification of TuMV VLP segments.** In panels **a-f** the cryoEM maps for the six classes calculated after classification of the total data set are depicted. The 3D unsupervised classification was performed in Relion2 and during the process no symmetry was imposed. The panels include the percentages of particles in each class.


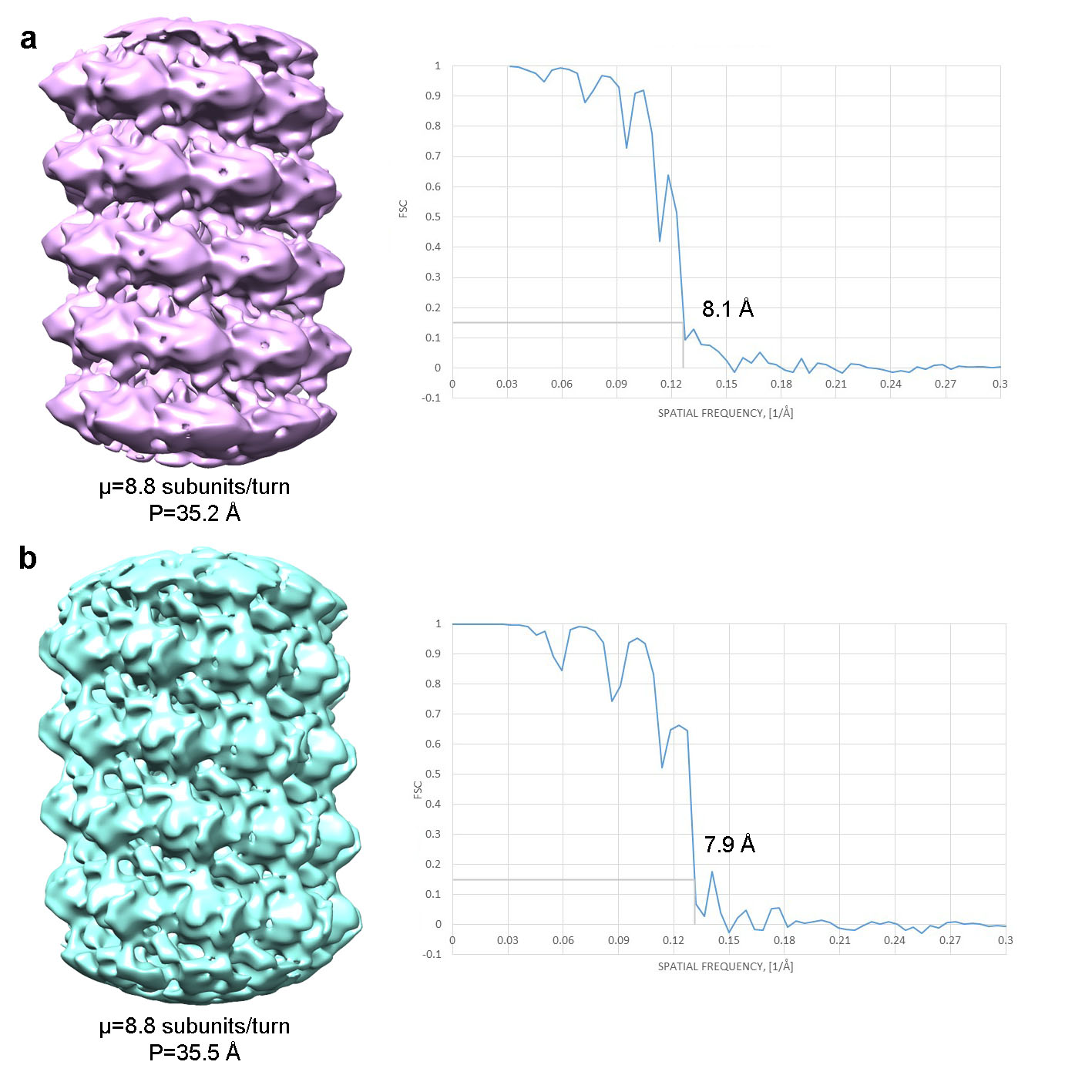


**Supplementary Fig.4 Refined cryoEM maps for two groups of TuMV VLPs. a** and **b** panels show respectively renderings for the 3D maps calculated for classes 1 and 3 after classification of the total set of VLP segments into six classes (shown in Supplementary Fig.3). The FSCs corresponding to each refinement are also shown, with estimated resolutions of about 8 Å. Refined parameters of helical symmetry are included in the panels.


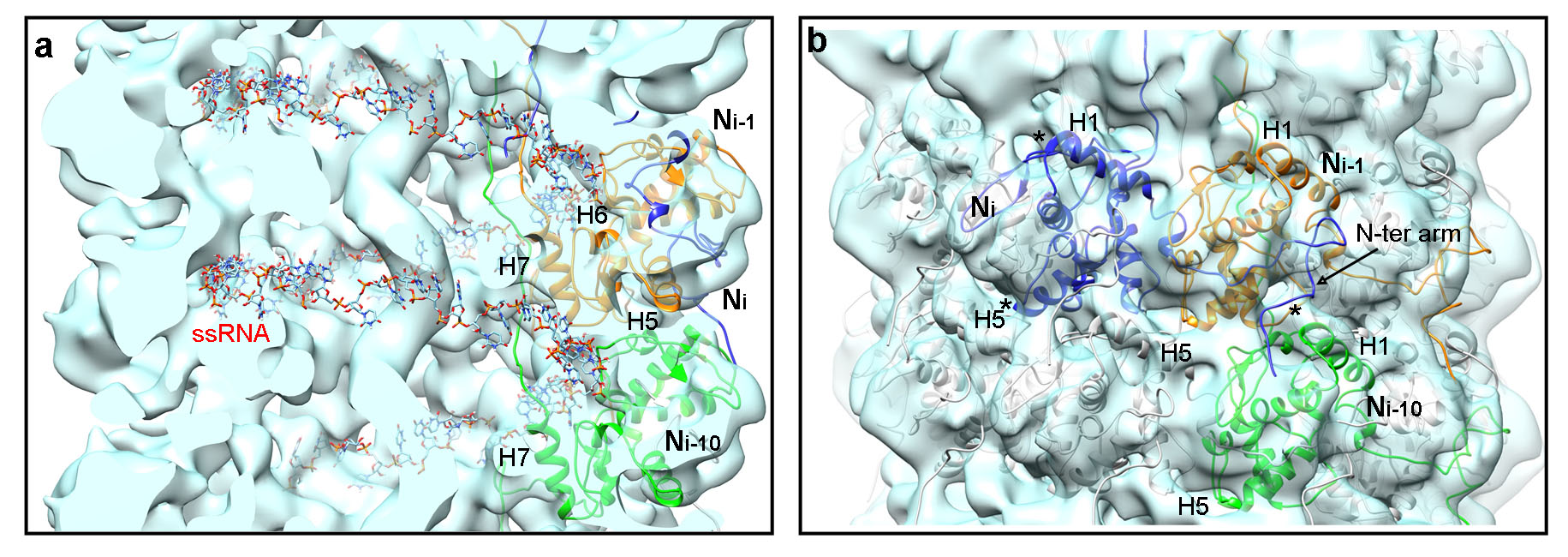


**Supplementary Fig.5 Structure of VLPs. a**, Cut-away rendering of the cryoEM map for class 3 of TuMV VLP. Representations of atomic models for several TuMV CPs and the ssRNA are shown. After rigid body fitting of the coordinates from TuMV virion, the ssRNA runs in an empty channel. **b**, The fitted coordinates for the multimer of TuMV CPs are seen inside the semitransparent map for class 3 of TuMV VLP. Regions of the atomic models that lie outside the density are labeled with asterisks in subunit N_i_. In the panels some α-helices of the atomic structure for TuMV CP are labeled.

**Supplementary Movie M1 Structural heterogeneity of TuMV virions.** The movie generated in Chimera contains the display of the volume series from the three classes of TuMV virions after classification. The animation shows that regions of the virus have some freedom to stretch and shrink.
